# GSTP1 Ile105Val Polymorphism and Prostate Cancer Risk: Evidence from a Meta-Analysis

**DOI:** 10.1371/journal.pone.0071640

**Published:** 2013-08-19

**Authors:** Bingbing Wei, You Zhou, Zhuoqun Xu, Jun Ruan, Huan Cheng, Ming Zhu, Qiang Hu, Ke Jin, Zhiqiang Yan, Deqi Zhou, Feng Xuan, Hongyi Zhou, Zhirong Wang, Xing Huang, Qiang Wang

**Affiliations:** 1 Department of Urology, Affiliated Wuxi People’s Hospital, Nanjing Medical University, Wuxi, China; 2 Minerva Foundation Institute for Medical Research, Biomedicum 2U, Helsinki, Finland; 3 Department of Urology, First Affiliated Hospital of Nanjing Medical University, Nanjing, China; University of Navarra, Spain

## Abstract

**Background:**

Glutathione S-transferase P1 (GSTP1) is thought to be involved in the detoxification of reactive carcinogen metabolites. Numerous epidemiological studies have evaluated the association of GSTP1 Ile105Val polymorphism with the risk of prostate cancer. However, the results remain inconclusive. To derive a more precise estimation, a meta-analysis was performed.

**Methodology/Principal Findings:**

A comprehensive search was conducted to identify the eligible studies. We used odds ratios (ORs) with 95% confidence intervals (CIs) to assess the strength of the relationship. The overall association was not significant (Val/Val vs. Ile/Ile OR = 1.06, 95% CI = 0.90–1.25, *P* = 0.50; Val/Val vs. Val/Ile+Ile/Ile: OR = 1.07, 95% CI = 0.91–1.25, *P* = 0.44). In subgroup analyses by ethnicity and prostate cancer grade, the similar results were observed. However, in stratified analysis by clinical stage, we found a significant association with low-stage prostate cancer (Val/Val vs. Ile/Ile: OR = 2.70, 95% CI = 1.73–4.22, *P*<0.001; Val/Val vs. Val/Ile+Ile/Ile: OR = 2.14, 95% CI = 1.38–3.33, *P* = 0.001). Moreover, there was no statistically significant evidence of multiplicative interactions neither between the GSTP1 Ile105Val polymorphism and GSTM1, nor between smoking status and GSTP1 on prostate cancer risk.

**Conclusions:**

This meta-analysis showed that GSTP1 Ile105Val polymorphism might not be significantly associated with overall prostate cancer risk. Further stratified analyses showed a significant association with low-stage prostate cancer.

## Introduction

Prostate cancer is the sixth most common cancer in the world, the third most common cancer in men, and the most common cancer in men in Europe, North America, and some parts of Africa [1]. In the United States, it is the second leading cause [2].http://en.wikipedia.org/wiki/Prostate_cancer. Several risk factors for prostate cancer have been identified, such as age, hormones, dietary factors and so on [1]. However, the definitive cause has yet to be elucidated [3]. Current studies suggest that both genetic and environmental factors may influence the pathogenesis of prostate cancer [4]. Numerous studies have shown that glutathione S-transferases (GSTs) are involved in the development of different cancers [5].

Glutathione-S-transferases (GSTs) are phase II enzymes which have been thought to be responsible for catalyzing the biotransformation of multiple electrophilic compounds [6]. It suggests that GSTs have a vital role in the detoxification of activated metabolites of procarcinogens produced by phase I reactions. In humans, eight gene families encode the cytosolic soluble GSTs namely, alpha (GSTA), mu (GSTM), theta (GSTT), pi (GSTP), sigma (GSTS), zeta (GSTZ), kappa (GSTK) and Omega (GSTO) [7]. Among them, several classes of GST enzymes, such as Pi, Alpha, Mu, and Theta, are expressed in prostate tissue, with Pi being the most abundant [8]. The polymorphisms in GSTM1 and GSTT1 are due to homozygous genetic deletions, which could result in absence of the GST enzyme activity, and might increase cancer susceptibility [6]. In contrast to most cancers [9], prostate cancer is associated with marked downregulation of GSTP1. GSTP1 may play a vital role in the development of prostate cancer [10], [11].

GSTP1 is thought to play an important role in susceptibility to prostate cancer. In prostate tissue, it is predominantly expressed in the basal layer of the normal prostate epithelium. Although these normal prostate secretory cells do not routinely express GSTP1, they still retain the capability to express this enzyme [12]. It is found that GSTP1 expression is increased in secretory cells of atrophic prostate epithelium, suggesting that the enzyme remains inducible in this cellular compartment. In addition, its expression is markedly diminished in an overwhelming majority of prostate cancer and prostatic intraepithelial neoplasia (PIN) specimens [13]–[15]. It is speculated that the early loss of GSTP1 function may lead to increased vulnerability to oxidant and heterocyclic amine carcinogens which are implicated in prostate carcinogenesis. Hence, it is possible that the heritable difference in GSTP1 function, due to GSTP1 genetic polymorphism, might be associated with prostate carcinogenesis.

A single nucleotide polymorphism (SNP) in the coding sequence at codon 105 (Ile105Val) of GSTP1 was reported to lower enzymatic activity in vitro [16]. It appears that the decreased detoxification capacity by reduced enzymatic activity may increase susceptibility to prostate cancer. In recent years, the association of GSTP1 Ile105Val polymorphism with prostate cancer risk has been extensively investigated [17]–[43]. One study showed that the frequency of GSTP1 Val/Val genotype was 14.3% in prostate cancer cases compared with 2.4% in controls, providing a notion that there is a significant association (OR: 3.72, 95% CI: 1.67–5.65; *P* = 0.002) [44]. Lavender et al. [17] observed a moderately significant association among men (OR = 1.56; 95%CI = 0.95–2.58; *P* = 0.049) and further confirmed it by MDR (multifactor dimensionality reduction) permutation testing (*P* = 0.001). However, other studies [18], [42] did not show significant results. In addition, GSTM1 have been extensively investigated in the last years. The GSTP1-GSTM1 interaction was also investigated in several studies [22], [24], [26], [31].

On the whole, the results about the association between GSTP1 Ile105Val polymorphism and prostate cancer risk were conflicting and inconclusive. To derive a more precise estimation, we performed a meta-analysis.

## Materials and methods

### Identification and Eligibility of Relevant Studies

PubMed (1956 to 20 June 2013), EMBASE (1974 to 20 June 2013), HuGENet (2000 to 20 June 2013), and Chinese National Knowledge Infrastructure (CNKI) (1978 to 20 June 2013) database searches were performed using the following terms: “GSTP1”, “polymorphism”, and “prostate”. The references of the retrieved articles were also screened. In case of studies with overlapping data, we selected the study with the largest number of subjects. The studies included in the meta-analysis should meet the following criteria: (a) evaluate the association of GSTP1 Ile105Val polymorphism with prostate cancer risk published in English or Chinese language, (b) use a case-control design, (c) provide the sample size, distribution of alleles, genotypes or other information that contribute to infer study characteristics, and (d) the distribution of GSTP1 genotypes in controls being consistent with Hardy-Weinberg equilibrium (HWE).

### Data Extraction

Two authors independently collected the information and reached consensus on all items. The following characteristics were extracted from eligible studies: name of first author, year of publication, country, ethnicity, numbers of genotyped prostate cancer cases and controls. Different ethnic descents were categorized as Caucasians, Asians, and Africans. If a study did not state the ethnic descendent or was not possible to separate participants according to their phenotype, the group [42] was termed “mixed ethnicity”.

Two studies [30], [39] only provided the information on genotypes as “Val/Val+Ile/Val” and “Ile/Ile” without details, so we could only calculate the odds ratio (OR) for the dominant genetic model. In subgroup analyses according to smoking status and GSTM1 null/present genotypes, the studies [22], [24]–[26], [30], [31], [42], [43] provided the information of genotypes as “Ile/Ile” and “Val/Val+Ile/Val”, and we calculated the OR for the dominant genetic model.

### Statistical Analysis

The strength of the association between GSTP1 Ile105Val polymorphism and prostate cancer risk was measured by ORs with 95% confidence intervals (CIs). We first estimated the effects of the Val/Val and Val/Ile genotypes on the risk of prostate cancer, compared with the wild-type Ile/Ile homozygote, then evaluated the effects of “Val/Val+Val/Ile vs. Ile/Ile” and “Val/Val vs. Val/Ile+Ile/Ile” on the risk, in dominant and recessive models, respectively. Subgroup analyses were also performed based on clinical stage, grade, ethnicity, smoking status, and GSTM1 null/present genotype.

Between studies heterogeneity was evaluated with the Q test based on the Chi-square distribution (*P*<0.10 was considered significant) [45], [46]. In case of heterogeneity, the random effects model was used to calculate the pooled OR [47], whereas the fixed effects model was used in its absence [48]. A sensitivity analysis was conducted to assess the stability of the results. For the control group of each study, the observed genotype frequency was assessed for HWE using the Pearson chi-square test; *P*<0.05 was considered significant. Funnel plots and Egger’s linear regression test were used to evaluate publication bias [49].

All statistical analyses were performed by using STATA (version 11.0; Stata Corporation, College Station, TX).

## Results

### Study Characteristics

**Figure 1:**
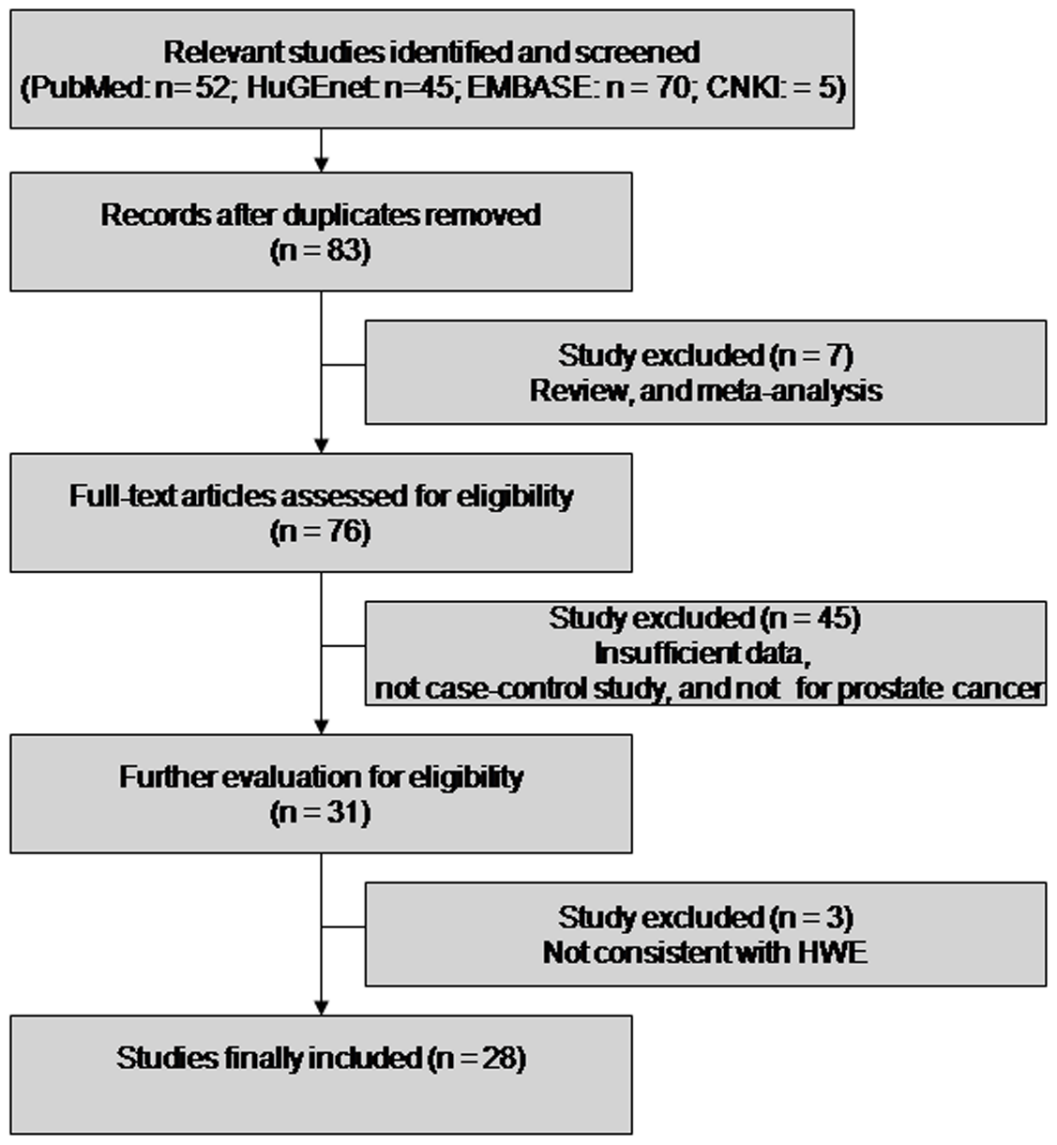
Flow chart of study selection based on the inclusion and exclusion criteria.

Publications were retrieved based on the search criteria. Study selection process was shown in Figure 1. Among them, the distribution of GSTP1 genotypes in controls was not consistent with HWE for the GSTP1 polymorphism in 2 studies [44], [50]. In the study by Agalliu et al [18], the distribution of GSTP1 genotypes among controls was not in agreement with the HWE among Africans. These studies were excluded from the meta-analysis. Finally, a total of 28 case-control studies involving 6,790 cases and 7,375 controls were included. The characteristics of these studies are summarized in Table S1. There were 8 studies on subjects of Asian, 17 of Caucasian, 2 of African, and 1 of mixed ethnicity. Prostate cancer was confirmed histologically or pathologically in all studies. Detailed information about prostate cancer stage and grade (Gleason scores) were shown in seven studies [20], [21], [24], [26], [27], [33], [39]. The data on smoking status and GSTM1 null/present genotypes was available in four studies [25], [30], [42], [43].

### Quantitative Synthesis

GSTP1 105Val allele frequencies in the cases and controls were calculated. The frequency of the 105Val allele was 30.12% (95%CI = 26.87–33.38%) in the cases, which was not significantly higher than that controls (29.17%; 95%CI = 26.02–32.33%, P = 0.67).

**Figure 2:**
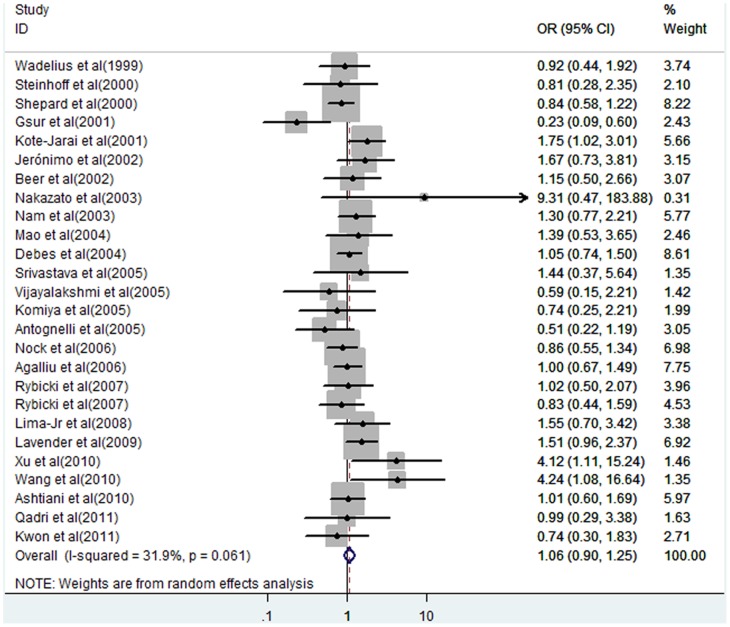
Forest plot of prostate cancer risk associated with GSTP1 Ile105Val polymorphism (for Val/Val vs. Ile/Ile). The squares and horizontal lines correspond to the study-specific OR and 95% CI. The area of the squares reflects the weight (inverse of the variance). The diamond represents the summary OR and 95% CI.

We performed a meta-analysis of the GSTP1 Ile105Val polymorphism overall, and in subgroups of ethnicities, clinical stage, and grade (Gleason scores) under various genetic models (Table S2). Overall, we did not find a significant association of Ile105Val polymorphism with the risk of prostate cancer (Val/Val vs. Ile/Ile: OR = 1.06, 95% CI = 0.90–1.25, *P* = 0.50; Val/Ile vs. Ile/Ile: OR = 1.02, 95% CI = 0.89–1.16, *P* = 0.83; Val/Val+Val/Ile vs. Ile/Ile: OR = 1.03, 95% CI = 0.91–1.16, *P* = 0.63; Val/Val vs. Val/Ile+Ile/Ile: OR = 1.07, 95% CI = 0.91–1.25, *P* = 0.44; Table S2, Figure 2). However, significant association of Ile105Val polymorphism was identified with low-stage prostate cancer (Val/Val vs. Ile/Ile: OR = 2.70, 95% CI = 1.73–4.22, *P*<0.001; Val/Val vs. Val/Ile+Ile/Ile: OR = 2.14, 95% CI = 1.38–3.33, *P* = 0.001; Table S2), but not with high-stage (Val/Val vs. Ile/Ile: OR = 1.57, 95% CI = 0.76–3.23, *P* = 0.22; Val/Val vs. Val/Ile+Ile/Ile: OR = 1.40 95% CI = 0.69–2.85, *P* = 0.35; Table S2). In addition, there was no evidence supporting that the Ile105Val polymorphism was significantly associated with prostate cancer risk in patients with high- and low-grade cancer (Table S2).

In subgroup analyses by ethnicities, the Ile105Val polymorphism was not associated with prostate cancer risk in Caucasians and Asians (Table S2).

### Cumulative Meta-analysis

In this study, a cumulative meta-analysis was also performed, in which the studies were added one at a time according to year of publication. We found that the pooled ORs did not change according to year of publication (data not shown).

### GSTP1- GSTM1 and GSTP1-smoking Interactions

The gene-gene and gene-environment interactions were assessed in our analysis. GSTM1 null/present genotypes have been identified [10], [51]. When the data were analyzed in subgroups of subjects stratified by GSTM1 null/present genotypes, we found that the Ile105Val polymorphism was not significantly associated with prostate cancer risk among patients with GSTM1 null genotype (Val/Val+Val/Ile vs. Ile/Ile: OR = 1.11 95% CI = 0.75–1.65, *P* = 0.56; Table S2), and among patients with GSTM1 present genotype (Table S2).

When the data were analyzed in subgroups of subjects stratified by smoking status, we did not find that GSTP1 Ile105Val polymorphism was significantly associated with prostate cancer risk among smokers (Val/Val+Val/Ile vs. Ile/Ile: OR = 0.90, 95% CI = 0.68–1.18, *P* = 0.43; Table S2), and among non-smokers (Table S2). The results were confirmed by logistic regression analysis and multifactor dimensionality reduction (MDR) method (data not shown).

### Test of Heterogeneity

The heterogeneity was reckoned between each of the studies using the Q-test. Overall, the significant heterogeneity was detected (Val/Val vs. Ile/Ile: *P*_heterogeneity = _0.06; Val/Ile vs. Ile/Ile: *P*_heterogeneity_ <0.01; Val/Val+Val/Ile vs. Ile/Ile: *P*_heterogeneity_ <0.01; Val/Val vs. Val/Ile+Ile/Ile: *P*_heterogeneity = _0.08). In stratified analyses by clinical stage, significant heterogeneity was observed for low-stage prostate cancer under one genetic model (Val/Ile vs. Ile/Ile: *P*_heterogeneity = _0.001). In stratified analyses by smoking status and GSTM1 null/present genotypes, there was no evidence of heterogeneity.

### Sensitivity Analysis

In the sensitivity analysis, the influence of each study on the pooled OR was examined by repeating the meta-analysis while omitting each study, one at a time. This procedure confirmed the stability of the overall result (data not shown).

### Publication Bias

**Figure 3:**
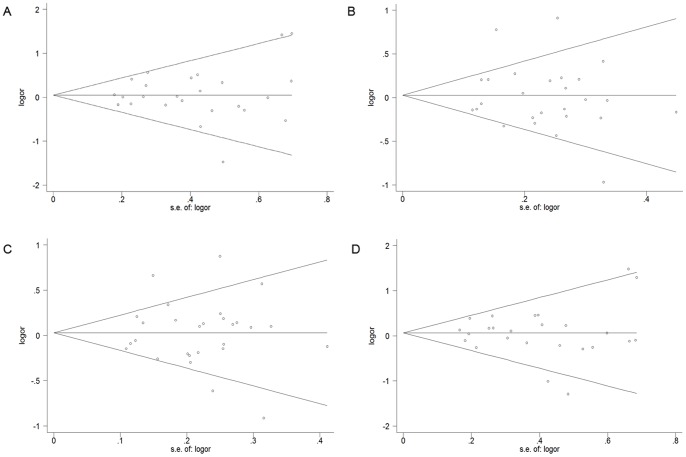
Begg’s funnel plots for publication bias test on studies assessing GSTP1 Ile105Val polymorphism and prostate cancer risk (A, Val/Val vs. Ile/Ile; B, Val/Ile vs. Ile/Ile; C, Val/Val vs. Val/Ile+Ile/Ile; D, Val/Val+Val/Ile vs. Ile/Ile). Each circle represents as an independent study for the indicated association.

Begg’s funnel plot and Egger’s test were conducted to assess publication bias. The shape of funnel plots did not show any evidences of asymmetry. The Egger’s test did not provide any statistical evidence of funnel plot symmetry (Val/Val vs. Ile/Ile: *P*_ = _0.82; Val/Ile vs. Ile/Ile: *P*_ = _0.69; Val/Val+Val/Ile vs. Ile/Ile: *P*_ = _0.98; Val/Val vs. Val/Ile+Ile/Ile: *P*_ = _0.90, Figure 3).

## Discussion

The present meta-analysis, including 6,790 prostate cancer cases and 7,375 controls, explored the association of one potentially functional polymorphism in the GSTP1 gene and prostate cancer risk. Overall, the significant association of GSTP1 Ile105Val polymorphism with prostate cancer risk was not detected. Moreover, the association was not significant in the subgroups according to ethnicity and prostate cancer grade. Reactive oxygen species generated from cigarette smoke are commonly thought to induce the formation of modified bases and single-strand breaks [52]–[54]. Accumulation of mutations in critical oncogenes and tumour suppressor genes promotes cancer. However, the results in our study does not support that smoking modifies the effect of GSTP1 Ile105Val polymorphism on prostate cancer risk. It is likely that GSTP1 enzyme activity in prostate cancer tissues has been reduced. The activity reduction may be due to hypermethylation of the CG island in the promoter region of the GSTP1 gene. Hence, it is speculated that the enzymatic differences due to the Ile105Val polymorphism might be not enough to affect overall prostate carcinogenesis and the roles of smoking. In addition, the influence of the genetic variant may be masked by the presence of other as-yet unidentified causal genes involved in prostate cancer. Further studies are warranted to define the etiology of this phenomenon.

Meta-analysis is a useful strategy for elucidating genetic factors in prostate cancer [55], [56]. GSTP1, which is involved in the detoxification of carcinogenic polycyclic aromatic hydrocarbons, has been investigated extensively in relation to different types of cancer, such as breast cancer [57], [58], bladder cancer [59], [60], oesophageal cancer [61], [62], and so on [5]. Previous results of the studies on the association of GSTP1 Ile105Val polymorphism with prostate cancer risk were inconclusive. These inconsistent results may be due to a small effect of the Ile105Val polymorphism on prostate cancer risk or the relatively low statistical power of the published studies. Although a meta-analysis has been published [63], the conclusion in our updated meta-analysis might be more convincing. In the meta-analysis [63], only 13 articles was included. However, in our meta-analysis, a total of 28 eligible studies were included which might lead to more convincing conclusions. In addition, gene-gene and gene-environment interactions were also investigated in our meta-analysis. Hence, we performed a meta-analysis for combining the results of various studies, and explaining their diversity. The results in the meta-analysis were more convincing.

In the meta-analysis, we did not find the significant association of Ile105Val polymorphism with overall prostate cancer risk. In the meta-analysis by Zhao et al [64], little evidence was found for the association between GSTP1 Ile105Val polymorphism and hepatocellular carcinoma risk. In another meta-analysis by Lang et al [65], the significant association between the GSTP1 Ile105Val polymorphism and the risk of head and neck cancers was not detected. In addition, the insignificant association of the Ile105Val polymorphism with thyroid cancer was also found in the meta-analysis by Li et al [66]. Hence, the overall conclusion in our study was consistent with that in other studies. However, we identified that the Ile105Val polymorphism was significantly associated with low-stage prostate cancer. This result suggested that enzymatic differences due to different GSTP1 genotypes played a pivotal role, and might influence the early phases of prostate cancer. The role of GSTP1 Ile105Val polymorphism might be specific according to low or high-stage of prostate cancer. Given the important functions of GSTP1 gene, it is biologically plausible that GSTP1 Ile105Val polymorphism may modify the risk of low-stage prostate cancer. Further experiments are needed to elucidate the difference.

GSTP1 was thought to be involved in detoxification of epoxides from carcinogenic polycyclic aromatic hydrocarbons and combination of exposure to cigarette smoking. Lack of GSTP1 activity might increase the burden with ultimate carcinogenic epoxides. In the stratified analyses by smoking status, the significant relationships were neither found among non-smokers nor smokers. It suggested that smoking might not significantly modify the effect of GSTP1 polymorphism on the risk of prostate cancer. However, the results on undetected effects should be interpreted with caution because of a relatively small sample size included in the subgroup.

Several limitations should be addressed in the meta-analysis. First, limited data restricted our further evaluation of potential GSTP1-GSTT1 interaction. Second, the sample size on the African populations was relatively small. Third, our results were based on the unadjusted evaluation. In order to provide a more precise estimation on the basis of adjustment for the confounders, well designed studies taking the potential confounders such as age into account are warranted. Forth, limited data in prospective case-control studies were included.

In summary, this meta-analysis provided evidence that the Ile105Val polymorphism was not related to overall prostate cancer risk. In subgroup analyses, the significant association of GSTP1 105Val allele with low-stage prostate cancer risk was observed. However, more sophisticated GSTP1–GSTT1 interaction should be considered for future experimental design, which would allow a comprehensive understanding of the association between GSTP1 Ile105Val and prostate cancer risk.

## Supporting Information

Table S1 **Characteristics of studies included in the meta-analysis.**
(DOC)

Table S2 **Stratified analyses of the GSTP1 Ile105Val polymorphism on prostate cancer risk.**
(DOC)
