# Supplementary material for: GSTP1 Ile105Val Polymorphism and Prostate Cancer Risk: Evidence from a Meta-Analysis
Source: PLoS One. 2013 Aug 19;8(8):e71640. doi: 10.1371/journal.pone.0071640 (PMC3747220; doi:10.1371/journal.pone.0071640)
Supplement: Table S1 — Characteristics of studies included in the meta-analysis. (DOC) [file pone.0071640.s001.doc]

Table S1. Characteristics of studies included in the meta-analysis.

| Author [reference] | Year | Country | Ethnicity | Cases | | |  | Controls | | |  |
| --- | --- | --- | --- | --- | --- | --- | --- | --- | --- | --- | --- |
|  |  |  |  | Ile/Ile | Ile/Val | Val/Val |  | Ile/Ile | Ile/Val | Val/Val | *P*_HWE_ |
| Autrup et al [30] | 1999 | <<Denmark> > | Caucasian | 72 | <81^a>^ | NA |  | 121 | <167 ^a>^ | NA | NA |
| Wadelius et al [35] | 1999 | <<Sweden> > | Caucasian | 87 | 67 | 17 |  | 80 | 51 | 17 | 0.05 |
| Steinhoff et al [31] | 2000 | <<Germany> > | Caucasian | 47 | 38 | 6 |  | 70 | 46 | 11 | 0.39 |
| Shepard et al [40] | 2000 | <<USA> > | Caucasian | 290 | 244 | 56 |  | 365 | 354 | 84 | 0.89 |
| Gsur et al [28] | 2001 | <<Austria> > | Caucasian | 90 | 51 | 6 |  | 65 | 57 | 19 | 0.26 |
| Kote-Jarai et al [36] | 2001 | <<UK> > | Caucasian | 117 | 115 | 41 |  | 140 | 105 | 28 | 0.21 |
| Jerónimo et al [34] | 2002 | <<Portugal> > | Caucasian | 45 | 55 | 16 |  | 61 | 67 | 13 | 0.37 |
| Beer et al [38] | 2002 | <<USA> > | Caucasian | 51 | 45 | 13 |  | 63 | 69 | 14 | 0.43 |
| Nakazato et al [26] | 2003 | <<Japan> > | Asian | 57 | 21 | 3 |  | 76 | 29 | 0 | 0.1 |
| Nam et al [29] | 2003 | <<Canada> > | Caucasian | 227 | 225 | 31 |  | 286 | 232 | 30 | 0.05 |
| Kidd et al [39] | 2003 | <<USA> > | Caucasian | 92 | <78 ^a>^ | NA |  | 95 | <73 ^a>^ | NA | NA |
| Mao et al [43] | 2004 | <<USA> > | Caucasian | 56 | 56 | 10 |  | 70 | 56 | 9 | 0.62 |
| Debes et al [41] | 2004 | <<USA> > | Caucasian | 369 | 414 | 131 |  | 184 | 236 | 62 | 0.31 |
| Srivastava et al [22] | 2005 | <<India> > | Asian | 46 | 77 | 4 |  | 83 | 56 | 5 | 0.23 |
| Vijayalakshmi et al [24] | 2005 | Indian | Asian | 49 | 22 | 4 |  | 43 | 51 | 6 | 0.07 |
| Komiya et al [25] | 2005 | <<Japan> > | Asian | 143 | 39 | 5 |  | 212 | 69 | 10 | 0.15 |
| Antognelli et al [33] | 2005 | <<Italy> > | Caucasian | 172 | 204 | 8 |  | 220 | 120 | 20 | 0.5 |
| Nock et al [37] | 2006 | <<USA> > | Caucasian | 175 | 222 | 42 |  | 207 | 214 | 58 | 0.81 |
| Agalliu et al [18] | 2006 | <<USA> > | Caucasian | 249 | 245 | 64 |  | 226 | 239 | 58 | 0.66 |
| Rybicki et al [19] | 2007 | <<USA> > | African | 82 | 146 | 46 |  | 29 | 59 | 16 | 0.12 |
| Rybicki et al [19] | 2007 | <<USA> > | Caucasian | 157 | 164 | 42 |  | 53 | 70 | 17 | 0.4 |
| Lima-Jr et al [42] | 2008 | <<Brazil> > | Mixed | 65 | 38 | 22 |  | 55 | 33 | 12 | 0.06 |
| Wang et al [21] | 2008 | <<China> > | Asian | 41 | 31 | 9 |  | 58 | 29 | 3 | 0.79 |
| Lavender et al [17] | 2009 | <<USA> > | African | 55 | 85 | 50 |  | 186 | 274 | 112 | 0.54 |
| Xu et al [20] | 2010 | <<China> > | Asian | 68 | 23 | 12 |  | 70 | 30 | 3 | 0.92 |
| Ashtiani et al [32] | 2011 | <<Germany> > | Caucasian | 125 | 95 | 28 |  | 216 | 228 | 48 | 0.28 |
| Qadri et al [23] | 2011 | <<India> > | Asian | 26 | 17 | 7 |  | 22 | 17 | 6 | 0.36 |
| Kwon et al [27] | 2011 | <<Korea> > | Asian | 117 | 42 | 7 |  | 209 | 101 | 17 | 0.3 |

NA, not available; a, genotypes as “Ile/Val + Val/Val”.
