# Supplementary material for: GSTP1 Ile105Val Polymorphism and Prostate Cancer Risk: Evidence from a Meta-Analysis
Source: PLoS One. 2013 Aug 19;8(8):e71640. doi: 10.1371/journal.pone.0071640 (PMC3747220; doi:10.1371/journal.pone.0071640)
Supplement: Table S2 — Stratified analyses of the GSTP1 Ile105Val polymorphism on prostate cancer risk. (DOC) [file pone.0071640.s002.doc]

Table S2. Stratified analyses of the GSTP1 Ile105Val polymorphism on prostate cancer risk.

| Vaviables | N^a^ | Cases/  Controls |  | Val/Val vs. Ile/Ile | | |  | Val/Ile vs. Ile/Ile | | |  | Val/Val + Val/Ile vs. Ile/Ile | | |  | Val/Val vs. Val/Ile + Ile/Ile | | |
| --- | --- | --- | --- | --- | --- | --- | --- | --- | --- | --- | --- | --- | --- | --- | --- | --- | --- | --- |
|  |  |  |  | OR(95% CI) | *P* | *P*^b^ |  | OR(95% CI) | *P* | *P*^b^ |  | OR(95% CI) | *P* | *P*^b^ |  | OR(95% CI) | *P* | *P*^b^ |
| Total | 28 | 6790/7375 |  | 1.06(0.90-1.25) | 0.50 | 0.06 |  | 1.02(0.89-1.16) | 0.83 | <0.01 |  | 1.03(0.91-1.16) | 0.63 | <0.01 |  | 1.07(0.91-1.25) | 0.44 | 0.06 |
| Ethnicity^d^ |  |  |  |  |  |  |  |  |  |  |  |  |  |  |  |  |  |  |
| Caucasian | 17 | 5331/5394 |  | 0.98(0.85-1.13) | 0.76 | 0.12 |  | 1.05(0.89-1.23) | 0.58 | <0.01 |  | 1.03(0.90-1.18) | 0.68 | <0.01 |  | 0.98(0.82-1.17) | 0.83 | 0.09 |
| Asian | 8 | 870/1205 |  | 1.34(0.88-2.02) | 0.17 | 0.11 |  | 0.94(0.64-1.40) | 0.77 | <0.01 |  | 1.02(0.69-1.49) | 0.93 | <0.01 |  | 1.37(0.91-2.07) | 0.13 | 0.18 |
| African | 2 | 464/676 |  | 1.35(0.92-1.97) | 0.13 | 0.36 |  | 0.98(0.72-1.34) | 0.92 | 0.58 |  | 1.08(0.81-1.44) | 0.60 | 0.40 |  | 1.35(0.97-1.88) | 0.07 | 0.45 |
| Clinical stage |  |  |  |  |  |  |  |  |  |  |  |  |  |  |  |  |  |  |
| Low | 5 | 689/1050 |  | 2.70(1.73-4.22) | <0.001 | 0.76 |  | 1.23(0.65-2.33) | 0.53 | 0.001 |  | 1.36(0.83-2.21) | 0.22 | 0.001 |  | 2.14(1.38-3.33) | 0.001 | 0.41 |
| High | 5 | 235/1050 |  | 1.57(0.76-3.23) | 0.22 | 0.10 |  | 1.32(0.73-2.38) | 0.36 | 0.07 |  | 1.20(0.71-2.02) | 0.49 | 0.03 |  | 1.40(0.69-2.85) | 0.35 | 0.18 |
| Gleason score |  |  |  |  |  |  |  |  |  |  |  |  |  |  |  |  |  |  |
| Low | 6 | 346/893 |  | 1.84(0.88-3.86) | 0.11 | 0.24 |  | 0.87(0.59-1.30) | 0.50 | 0.15 |  | 1.05(0.76-1.43) | 0.76 | 0.20 |  | 2.02(0.97-4.21) | 0.06 | 0.41 |
| High | 6 | 287/893 |  | 1.29(0.32-5.28) | 0.72 | 0.07 |  | 0.74(0.43-1.27) | 0.28 | 0.07 |  | 0.71(0.42-1.19) | 0.19 | 0.03 |  | 1.13(0.55-2.32) | 0.73 | 0.13 |
| Smoking status |  |  |  |  |  |  |  |  |  |  |  |  |  |  |  |  |  |  |
| Non-smoker | 4 | 183/367 |  | NA^c^ | NA | NA |  | NA | NA | NA |  | 1.03(0.72-1.49) | 0.87 | 0.22 |  | NA | NA | NA |
| Smoker | 4 | 402/547 |  | NA | NA | NA |  | NA | NA | NA |  | 0.90(0.68-1.18) | 0.43 | 0.33 |  | NA | NA | NA |
| GSTM1 genotype |  |  |  |  |  |  |  |  |  |  |  |  |  |  |  |  |  |  |
| Null | 4 | 201/203 |  | NA | NA | NA |  | NA | NA | NA |  | 1.11(0.75-1.65) | 0.56 | 0.41 |  | NA | NA | NA |
| Present | 4 | 173/273 |  | NA | NA | NA |  | NA | NA | NA |  | 1.12(0.31-4.07) | 0.86 | <0.01 |  | NA | NA | NA |

a number of included studies.

b *P* value of Q-test for heterogeneity test.

c not available.

d the mixed ethnicity subgroup was not included because there is only one study with mixed ethnicity.
